# Association between parental separation, childhood trauma, neuroticism, and depression: a case control study

**DOI:** 10.3389/fpsyt.2023.1112664

**Published:** 2023-05-09

**Authors:** Simon Sanwald, GenEmo Research Group, Bernhard J. Connemann, Christian Montag, Markus Kiefer

**Affiliations:** ^1^Department of Psychiatry and Psychotherapy III, Ulm University, Ulm, Germany; ^2^Department of Molecular Psychology, Institute of Psychology and Education, Ulm University, Ulm, Germany

**Keywords:** stressful life event, childhood trauma, separation, divorce, personality, neuroticism, depression

## Abstract

**Background:**

Parental separation has been suggested to be associated with depression development in offspring. The new family constellation subsequent to separation could be associated with elevated scores of childhood trauma, shaping more emotionally instable personalities. This could ultimately be a risk factor for mood disorders and particularly the development of depression in life.

**Methods:**

To test this hypothesis, we investigated the associations between parental separation, childhood trauma (CTQ) and personality (NEO-FFI) in a sample of *N* = 119 patients diagnosed with depression and *N* = 119 age and sex matched healthy controls.

**Results:**

While parental separation was associated with elevated scores of childhood trauma, there was no association between parental separation and Neuroticism. Furthermore, in a logistic regression analysis, Neuroticism and childhood trauma were found to be significant predictors for depression diagnosis (yes/no), but not parental separation (yes/no).

**Conclusion:**

Parental separation might be associated with depression only indirectly *via* childhood trauma. Childhood trauma or Neuroticism seem more directly related to the development of depression. However, it is worthwhile to install prevention programs helping parents and children to cope with parental separation in order to minimize the impact of separation and associated stressors.

## 1. Introduction

In 2021, 51.5% of all divorced couples in Germany had minor children, which resulted in 121,800 minors who had to learn how to deal with this new life situation (1). Since several decades, scientists and clinical practitioners have been concerned with the effect of parental divorce or separation on mental wellbeing in children (2, 3). Previous meta-analyses found significant associations between parental divorce and (among other outcome variables) problems with psychological adjustment, social relations and lower mental wellbeing (4, 5). More recent meta-analyses found positive associations between parental divorce and depression (6, 7). However, the authors highlighted that out of nine studies included in the meta-analysis eight were based on self-report measures of depression, i.e. depression was not diagnosed by means of a face-to-face interview with a psychiatrist. For that reason, investigations of patients diagnosed with depression according to DSM criteria are warranted (7). According to these meta-analytic data, parental separation beyond being an adverse childhood event itself could be an economic proxy for a variety of other childhood adversities and thus an easily assessable marker for individuals at risk of developing depression (7).

Various factors associated with the parental separation process could negatively affect development and emotional wellbeing of the offspring: Separation has been shown to be associated with psychological as well as economic hardships for parents (8), and ultimately with the development of mental disorders like depression (9). Thus, parents can be too caught up in their own emotions to be able to adequately care for their children's emotional needs, which might result in elevated levels of emotional abuse or neglect (10). Further, emotional distress in newly separated parents might also lead to a higher probability of physical abuse of their offspring (10, 11). Childhood adversities – especially emotional abuse –, in turn, are one of the best confirmed risk factors for depression development (12, 13). On the other hand, parental separation might also have positive consequences for children. For example, children with separated parents have been found to be more mature, have higher self-esteem, empathy and androgyny (more liberal sex-role self-concepts) than children with non-separated parents (14). Ultimately, the outcomes of parental separation for the child might depend on how parents cope with their separation and on parents' custody agreement (10).

To transfer their effect into adulthood, parental separation and childhood adversities should affect stable traits of an individual representing markers of an enduring liability to develop depression. In fact, childhood trauma is associated with maladaptive personality traits (15, 16). Accordingly, high Neuroticism – a trait defined as the tendency to experience negative emotions (17) – is considered a risk factor paving the way to the clinical phenotype of depression (18, 19). Therefore, it is surprising that the association between parental separation and offspring personality has only rarely been studied. Studies that investigated the influence of parental separation on children's psychological and personality development suggest the association with separation to be moderated by parents' characteristics like education or mental disorders (20, 21). However, parental separation in combination with other childhood adversities has been found to be positively related with Neuroticism in a previous study (22). Neuroticism, in turn, is strongly associated with elevated risk for depression development (23).

Therefore, we wanted to investigate the associations between parental separation during participants' childhood, maltreatment in childhood as well as Neuroticism in a sample of inpatients diagnosed with depression according to DSM-IV criteria and in matched healthy controls. Furthermore, we wanted to examine whether parental separation during childhood explains additional variance regarding depression diagnosis when childhood adversities as well as Neuroticism are controlled for. To this end, we analyzed data of *N* = 238 participants (*N* = 119 participants per group). Our sample (patients and controls) was taken from the database of the Ulm Gene Brain Behavior Project (UGBBP). Based on previous results (10, 11, 23), we assumed patients to report more childhood maltreatment, to report more often that parents have been separated and to score higher on Neuroticism as compared to healthy controls. Additionally, we hypothesized that parental separation is associated with elevated scores indicating emotional abuse, emotional neglect, physical abuse and Neuroticism. Since previous studies suggest depression diagnosis (24) as well as having separated parents (11) to be associated with higher levels of childhood maltreatment, we expected patients having separated parents to score significantly higher on childhood maltreatment than patients without separated parents or healthy controls independently of parental relationship status.

## 2. Materials and methods

### 2.1. Participants

Before matching, data of 162 patients suffering from Major Depressive Disorder (MDD) and 120 healthy control participants was taken from the database of the UGBBP comprising both patient and healthy control samples. We included all individuals of the database, who completed the childhood trauma questionnaire (CTQ, 25). Data partially overlapped with those of earlier studies with a focus on different research questions (26–28). Patients were recruited at the Department of Psychiatry and Psychotherapy III at Ulm University, Ulm, Germany and were diagnosed for MDD by a psychiatrist at admission to the hospital by means of the Structured Clinical Interview for DSM-IV (SCID-I) (29).

Healthy controls were recruited by postings in public areas and online advertisement. To ensure that controls did not suffer from any kind of mental illness, they underwent a diagnostic interview comprising the Mini-DIPS (30) and SCID-II (29). An additional exclusion criterion was a lifetime diagnosis of any kind of mental illness or any kind of past psychiatric inpatient treatment or psychotherapy. Data of 120 participants of the control sample met inclusion criteria and was further analyzed. All procedures performed in this study were approved by the ethics committee of Ulm University, Ulm, Germany and were in accordance with the 1964 Helsinki declaration and its later amendments. Informed consent of the participants was obtained after the nature of the procedures had been fully explained.

Both groups were administered the questionnaires described below. A semi-structured interview based on an in-house questionnaire was used to obtain sociodemographic variables. All participants answered the question of whether their parents are separated/divorced and indicated their age at separation of their parents. In the group of healthy controls, 1 male participant and in the group of patients with MDD, 6 men and 13 women did not answer one of these questions and were excluded from analyses with parental separation. We investigated parental separation before the age of 18 [as this age was used as cutoff in earlier studies, e.g., (31)]. Each of the *N* = 120 healthy control individuals was matched with one of the *N* = 162 inpatients using exact matching in case of sex and nearest neighbor matching (propensity score) in case of age (see Statistical Analyses). As a result, paired samples of *N* = 119 depressed inpatients and *N* = 119 healthy controls (86 females each) did not differ significantly in their age (controls: *M* = 32.45 years, *SD* = 11.97 years, range 18–63 years; patients: *M* = 32.42 years, *SD* = 12.40 years, range 18–57 years). For descriptive statistics, refer to Table 1.

**Table 1:** Descriptive statistics of the investigated variables for healthy controls and patients.

**Variable**	**Healthy controls**					**MDD**				
	***n***	***min***	***max***	***M***	***SD***	***n***	***min***	***max***	***M***	***SD***
Childhood Maltreatment total	119	25.00	85.00	34.24	9.65	119	25.00	114.00	49.79	18.63
EA	119	5.00	22.00	7.70	3.48	119	5.00	25.00	12.48	5.73
PA	119	5.00	16.00	5.66	1.62	119	5.00	23.00	7.66	3.97
SA	119	5.00	12.00	5.21	0.85	119	5.00	25.00	7.32	4.37
EN	119	5.00	23.00	9.08	4.09	119	5.00	25.00	13.65	5.44
PN	119	5.00	19.00	6.58	2.35	119	5.00	19.00	8.69	3.89
Depression severity	119	0.00	20.00	5.64	4.94	113	8.00	53.00	32.56	10.00
Neuroticism	119	1.33	4.42	2.57	0.68	118	2.42	4.92	4.03	0.57
Extraversion	119	2.08	4.67	3.49	0.52	118	1.17	4.17	2.60	0.60
Openness	119	2.17	4.67	3.45	0.52	118	1.92	4.45	3.19	0.57
Agreeableness	119	2.50	4.83	3.78	0.50	118	1.83	4.73	3.40	0.51
Conscientiousness	119	2.00	5.00	3.88	0.49	118	1.58	4.67	3.28	0.63

Mean (M), standard deviation (SD), emotional abuse (EA), physical abuse (PA), sexual abuse (SA), emotional neglect (EN), physical neglect (PN), Major Depressive Disorder (MDD).

### 2.2. Questionnaires

#### 2.2.1. Childhood trauma questionnaire

The short form of the Childhood Trauma Questionnaire (CTQ, (25)) is a self-report questionnaire comprising 28 items. It yields scores on five subscales of traumatic events [emotional abuse (EA), physical abuse (PA), sexual abuse (SA), physical neglect (PN) and emotional neglect (EN)] as well as a total score using a Likert scale format with a range from 1 (never true) to 5 (very often true). EA, PA, SA, PN and EN range from 5 to 25 and the total score ranges from 25 to 125. Internal consistencies were acceptable to excellent for all subscales in both groups, except for the low internal consistency of physical neglect in the group of healthy controls (Table 2).

**Table 2:** Internal consistencies for the investigated questionnaire data.

	**Healthy Control**	**MDD**
	α	α
Childhood maltreatment total	0.89	0.94
EA	0.84	0.89
PA	0.71	0.84
SA	0.74	0.95
EN	0.90	0.92
PN	0.50	0.72
Depression severity	0.82	0.87
Neuroticism	0.88	0.81
Extraversion	0.78	0.80
Openness	0.72	0.71
Agreeableness	0.79	0.70
Conscientiousness	0.79	0.83

Same conventions as in Table 1.

#### 2.2.2. Beck depression inventory

Severity of depressive symptoms was explored using the Beck Depression Inventory [German version, BDI-II; (32)]. It is a self-assessment scale that contains 21 items, in which for each item ratings between 0 (not at all) and 3 (very intensive) are given depending on the symptom complaint. Thus, BDI-II scores range from 0 to 63. Internal consistency was good in both groups (Table 2).

#### 2.2.3. Neuroticism extraversion openness-five-factor inventory

The Neuroticism Extraversion Openness-Five-Factor Inventory (NEO-FFI, 17) assesses via 60 items the personality traits called Openness to Experience, Conscientiousness, Extraversion, Agreeableness, and Neuroticism. The items are answered on a five-point Likert scale ranging from strongly disagree (1) to strongly agree (5). Personality traits are means of the corresponding items. Higher scores represent higher expression of each of the personality dimensions. The German version of the NEO-FFI was administered (33). Internal consistencies are in Table 2 and were acceptable to good.

### 2.3. Statistical analysis

Statistical analysis was conducted using *R* [R (34)] with the packages *psych* (35) and *matchIt* (36). Additionally, we used *JASP* for ANOVAs [JASP (37)]. Matching was performed by calculating propensity scores for patients suffering from depression and healthy controls. Thereafter, each control participant was matched one inpatient of the same sex using the nearest neighbor method (36).

Afterwards, we compared frequencies of parental separation (as well as its interaction with sex) between patients and controls performing chi-squared tests. In addition, we examined group differences in CTQ total score and Neuroticism as well as group by parental separation interactions by means of two-way ANOVAs. In order to test the associations between childhood trauma and personality in our sample, we performed correlation analyses (Pearson; note that Spearman's correlation coefficients provided a similar result pattern; age and gender were not further controlled since they did not differ between groups). Last, we performed a logistic regression analysis with childhood trauma, Neuroticism as well as parental separation as predictors and group (patients with depressive disorder vs. healthy control participant) as dependent variable.

Statistical significance was determined at *p* < 0.05; all tests were two-tailed. False discovery rate (FDR) was controlled with the Benjamini-Hochberg method for correlation analyses (38).

## 3. Results

### 3.1. Differences in frequencies in parental separation

Groups did not significantly differ regarding the frequency of parental separation (controls: 87 no separation, 32 separation; patients: 66 no separation, 33 separation; χ^2^(1) = 1.07, *p* = 0.301). There were no significant differences in the frequency distribution of parental separation as a function of sex and group (healthy control group: men: 25 no separation, 8 separation; women: 62 no separation, 24 separation; MDD: men: 13 no separation, 14 separation; women: 53 no separation, 19 separation; χ^2^(3) = 4.92, *p* = 0.178).

### 3.2. Interactions between group and parental separation

**Figure 1:**
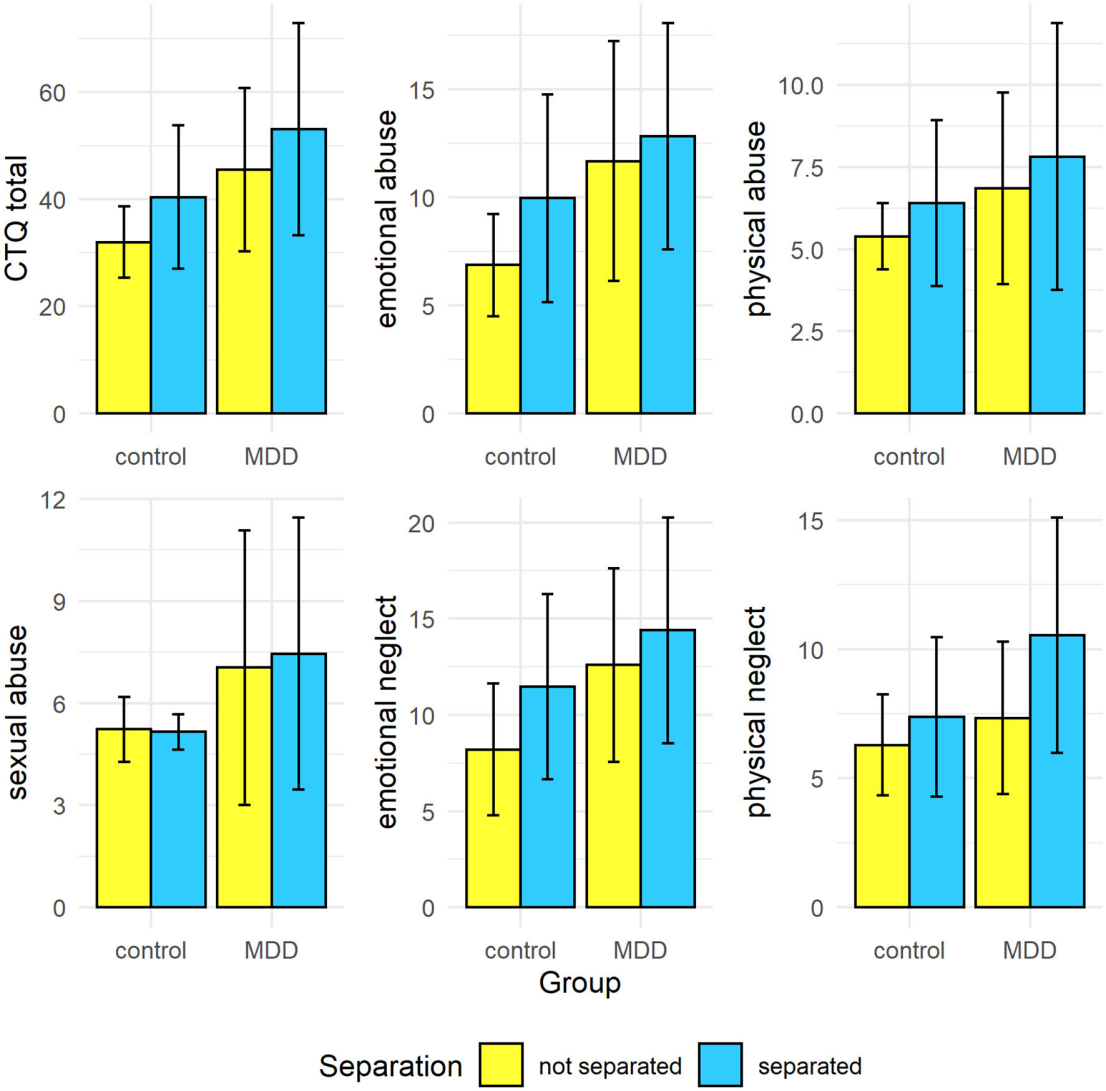
Means and SDs by group and parental separation regarding CTQ total and CTQ subscales.

The ANOVA with the total score of the CTQ revealed a significant main effect of group (controls vs. patients) [*F*_(1, 214)_ = 44.90, *p* < 0.001, *partial* η^2^ = 0.173] with patients showing higher scores and a significant main effect of parental separation with parental separation being associated with higher scores (not separated: *M* = 38.74, *SE* = 1.07; separated: *M* = 46.73, *SE* = 1.63; *F*_(1, 214)_ = 16.62, *p* < 0.001, *partial* η^2^ = 0.072). The interaction of group and parental separation was not significant [*F*_(1, 214)_ = 8.54, *p* = 0.824]. The investigation of subscales of the CTQ revealed a similar result pattern for all subscales with significant main effects, i.e., main effects for group and parental separation with patients scoring higher than controls and parental separation being associated with higher scores than no parental separation. There was one exception: For sexual abuse there was no significant main effect of parental separation [*F*_(1, 214)_ = 0.17, *p* = 0.685]. However, in case of physical neglect in addition to main effects of group and separation, there was a significant interaction between group and separation [*F*_(1, 214)_ = 5.91, *p* = 0.016, *partial* η^2^ = 0.027; Figure 1]. Patients with separated parents scored higher compared to all other participant groups (Tukey-HSD: other conditions vs. MDD with separated parents: all *p* < 0.001). Interestingly, controls with separated parents did not differ from controls with non-separated parents regarding physical neglect (Tukey-HSD: *p* = 0.282; Figure 1).

ANOVAs with personality dimensions as dependent variables all yielded the same result pattern: There was a significant main effect of group but no other effect – neither the main effect of separation nor the interaction between group and separation – was significant (all other *p*-values > 0.05). Compared to healthy controls, patients scored significantly higher on Neuroticism and significantly lower on all other personality dimensions [Neuroticism: *F*_(1, 214)_ = 247.49, *p* < 0.001, *partial* η^2^ = 0.536; Extraversion: *F*_(1, 214)_ = 105.30, *p* < 0.001, *partial* η^2^ = 0.330; Openness: *F*_(1, 214)_ = 9.29, *p* = 0.003, *partial* η^2^ = 0.042; Agreeableness: *F*_(1, 214)_ = 19.49, *p* < 0.001, *partial* η^2^ = 0.083; Conscientiousness: *F*_(1, 214)_ = 67.16, *p* < 0.001, *partial* η^2^ = 0.239).

### 3.3. Correlation analyses

There were significant positive associations between CTQ total, emotional abuse and emotional neglect and depression severity in both groups (Table 3). In addition, CTQ total as well as all subscales (except physical/sexual abuse in patients and physical abuse in healthy controls) were significantly negatively associated with Agreeableness in both groups.

**Table 3:** Pearson's correlation coefficients for associations between childhood trauma and personality dimensions in both groups.

**MDD**						
	**CTQ total**	**EA**	**PA**	**SA**	**EN**	**PN**
Depression severity	0.25^**^	0.36^***^	0.04	0.19^*^	0.24^*^	0.08
Neuroticism	0.12	0.20^*^	0.07	0.15	0.04	−0.02
Extraversion	0.02	−0.08	0.09	0.05	−0.02	0.07
Openness	0.09	0.18^*^	0.14	−0.01	0.08	−0.07
Agreeableness	−0.27^**^	−0.25^**^	−0.18	−0.11	−0.25^**^	−0.24^**^
Conscientiousness	−0.06	−0.12	−0.13	0.05	−0.03	0.00
**Healthy controls**						
Depression severity	0.25^**^	0.26^**^	0.15	0.06	0.21^*^	0.15
Neuroticism	0.10	0.17	0.04	0.06	0.03	0.07
Extraversion	−0.19^*^	−0.23^*^	−0.03	0.01	−0.21^*^	−0.07
Openness	−0.05	−0.06	0.01	0.14	−0.06	−0.08
Agreeableness	−0.44^***^	−0.41^***^	−0.16	−0.22^*^	−0.36^***^	−0.38^***^
Conscientiousness	0.11	0.04	0.05	0.00	0.12	0.14

EA, Emotional abuse, PA, Physical abuse, SA, sexual abuse, EN, emotional neglect; PN, physical neglect.

*** p_BH_ < .001,

** p_BH_ < .01,

* p_BH_ < .05.

Emotional abuse was significantly positively associated with Neuroticism in the group of patients.

CTQ total, emotional abuse and emotional neglect were significantly associated with Extraversion in healthy controls.

Taken together, the result pattern was very similar in both groups.

#### 3.3.1. Predicting depression diagnosis

A logistic regression model with group as dependent variable and separation, sex as well as their interaction as independent variables was not significantly better than an intercept-only model [χ^2^(3) = 1.22, *p* = 0.748]. However, adding the total score of childhood trauma as well as Neuroticism as independent variables resulted in a model that was significantly better than the intercept-only model [χ^2^(5) = 33.35, *p* < 0.001] with significant main effects of childhood trauma [*b* = 0.01, *SE* = 0.002, *t*_(212)_ = 3.44, *p* < 0.001] and Neuroticism [*b* = 0.36, *SE* = 0.02, *t*_(212)_ = 14.80, *p* < 0.001]. Neither main effects for separation or sex nor their interaction were significant predictors of group in this model (all *p* > 0.200).

## 4. Discussion

In the present study, we investigated whether parental separation before the age of 18 is associated with depression, a history of childhood trauma as well as higher Neuroticism. In addition, we examined whether parental separation is predictive of depression diagnosis above well-established risk factors like childhood trauma and Neuroticism.

Contrary to our expectations, parental separation was not associated with depression diagnosis. The absence of a significant association between parental separation and depression diagnosis resembles the heterogeneity of previous case control studies examining this relationship: There are studies reporting a significant relationship between depression and parental divorce vs. intact families (39, 40). On the other hand, there are studies unable to detect significant differences between offspring from separated parents vs. parents still in a relationship regarding depression (41, 42). However, power in our study may be too low to detect a small difference in frequencies of parental separation comparing patients suffering from depression to healthy controls.

As expected, depression as well as parental separation were associated with higher scores of childhood trauma. The main effect of group points toward the close and well established link between childhood trauma and depression risk potentially due to hypothalamus-pituitary axis (HPA axis) hyper(re-)activity (43). An explanation for the association between parental separation and higher childhood trauma (total and all subscales besides sexual abuse) could be that separation may bring challenges due to, for example, spatial and/or financial changes that may affect the parents in such a way that emotional devotion to the offspring suffers or even physical violence becomes more likely (10, 11). Conversely, separation is a result of conflict and thus conflict may have been higher in these families long before parental separation and parental separation could bring an improvement regarding the various forms of abuse and neglect (21). Since we used a cross-sectional design, we cannot draw causal conclusions or speculate on whether separation or the various forms of childhood trauma occurred earlier or later in time. In addition, we found a significant interaction of group and separation regarding physical neglect. Patients suffering from depression with separated parents indicated significantly higher scores of physical neglect compared to all other combinations of group and parental separation. Healthy controls with or without separated parents and patients without separated parents did not significantly differ from each other. These results could be interpreted before the background of separation associated depression of the caregiver, which has been shown to be associated with physical neglect of the prodigy (44). The “silent” types of childhood maltreatment (other than forms of abuse) like neglect were found to have an impact on depression development (45), which is why the interrelations of depression of the caregiver, physical neglect and offspring depression need further investigation.

Interestingly, in the ANOVAs with personality dimensions as dependent variables, parental separation was neither associated with Neuroticism nor with any other personality dimension. This is unexpected not only before the background of the positive association between childhood trauma and Neuroticism (16) but also since Neuroticism is thought to be moderately heritable (46, 47), and Neuroticism is negatively associated with relationships satisfaction (48, 49). Therefore, parental Neuroticism could be assumed to contribute to parental separation and may also be associated with offspring Neuroticism scores. Moreover, we found the expected association between parental separation and childhood trauma, and childhood trauma was significantly associated with personality. We found significant negative associations between the CTQ total score and Agreeableness in both samples. In addition, CTQ total score was significantly negatively associated with Extraversion in healthy controls. In both samples, emotional abuse and emotional neglect were negatively associated with Agreeableness. Furthermore, emotional abuse was significantly positively associated with Neuroticism in patients suffering from depression. These findings are in line with results of previous studies showing that emotional abuse was most pervasively related to personality (50) and that early maladaptive schemas like mistrust (expecting others to intentionally hurt, abuse, cheat, lie, etc.) or failure (believing that one has failed or will fail in areas of achievement), which are also strongly related with depression (51), are negatively associated with Agreeableness (52). Therefore, while childhood maltreatment has a plausible pathway to transfer its effect into a risk for developing depression, parental separation does not, at least according to our (correlational) results. In support of this interpretation, a model with parental separation as predictor for depression was not significantly better than an intercept-only model. Furthermore, parental separation did not explain a significant amount of variance in depression when adding it as predictor to a model that already included childhood maltreatment and Neuroticism. It has been postulated that an individual's genetic vulnerability in combination with stressful life events is associated with changes in epigenetic regulation of HPA axis associated genes (53). Alterations in the epigenetic landscape of such genes are associated with differences in perception and coping with stressors and ultimately may lead to depression development (54). Parental separation alone on the other hand might not be such a useful predictor of depression risk. It is possible that parental separation has diametrically opposite meanings depending on the individual and contextual factors before, during and after parental separation, which would explain the absence of significant group differences in personality regarding parental separation. Accordingly, on the one hand, one child may experience separation distress as a result of not being able to see one parent anymore or has parents that are too caught up in dealing with the separation themselves to adequately care for their child (10, 11). On the other hand, for another child separation may be the equivalent of a reduction of family conflict and even improve the relationship to the now separated parents.

Some limitations need to be considered: First, we analyzed cross-sectional data and can therefore not derive causal conclusions. Second, even though it is challenging to collect data of patients diagnosed with depression and thoroughly screened healthy controls, our sample might be too small to detect small differences in frequency distributions regarding group and parental separation. Therefore, we are in need of meta-analytic analysis of many studies with patients diagnosed with depression and mentally healthy individuals, something that – to our knowledge – is lacking so far. Third, there is a plethora of variables associated with parental separation, which also may be related to mental health of the child (e.g., parents' custody agreement, how parents cope with the separation themselves, financial resources, etc.) that we did not account for. The investigation of parents' assessment of their separation would provide valuable insights in the processes that unfold before and after their separation. Fourth, we did not assess how traumatic participants felt about the separation of their parents or about other contextual factors specifically in the time around the separation. This, however, could provide valuable insights as to what factors associated with parental separation promote depression vulnerability. Last, we used a self-report measure to assess childhood trauma. While there is the potential of recall bias in patients suffering from depression, studies investigating non-recall measurement childhood trauma show similar associations between childhood trauma and depression as compared to studies investigating self-reported trauma (55, 56).

Parental separation was not associated with depression in our case-control study of patients and healthy controls, while childhood trauma and Neuroticism were significantly associated with depression diagnosis. However, parental separation was significantly associated with elevated scores of childhood trauma. Therefore, it is worthwhile to install prevention programs helping parents and children to cope with this difficult situation whether it is by financial aid or mental healthcare. Since we did not find parental separation unlike other forms of childhood adversities to be associated with depression-associated personality traits, it may ultimately depend on the individual or situational characteristics whether parental separation is a relevant topic in depression development and whether it should be addressed in psychotherapy.

## Data availability statement

The raw data supporting the conclusions of this article will be made available by the authors, without undue reservation.

## Ethics statement

The studies involving human participants were reviewed and approved by the Ethikkommission der Universität Ulm. The patients/participants provided their written informed consent to participate in this study.

## Author contributions

SS, CM, MK, and the GenEmo Research group designed the present study. SS analyzed the data and wrote the first draft of the manuscript. CM and MK commented on and improved previous versions of the manuscript. All authors contributed to and approved the manuscript.

## GenEmo Research Group

Bernhard J. Connemann, Carlos Schönfeldt-Lecuona, Thomas Kammer.
